# The School Doctor and the Compulsion of Parents

**Published:** 1916-12-30

**Authors:** 


### THE SCHOOL DOCTOR.

### The School Doctor and the Compulsion of Parents.

A QUESTION of some interest to the general public is raised by the decision of certain local education authorities to use legal means to enforce the recommendations of the school medical officers on recalcitrant parents. Under the Children Act and kindred measures the legal powers at their command are considerable, but they have been little used. The very idea of intervention between parent and child on the part of a public official is repugnant to British instincts. It must also be remembered that parents are perfectly free to withdraw their children from medical inspection if they please, and the success of the school doctor's work depends ultimately on their goodwill. On the other hand, the school doctor has a clear duty to protect helpless children from the results of ignorance and neglect, and he must be prepared to act boldly and regardless of criticism on occasion.

It has occurred to many social observers that the huge mass of defects which remain untreated after detection at routine medical inspection affords an obvious field for drastic measures of coercion. But a closer analysis of the territorial distribution of these untreated ailments (which, according to Sir George Newman's latest report, amount to 40 per cent. of the whole number recorded) suggests that the real fault is often lack of opportunity rather than deliberate neglect. Districts where facilities for treatment are freely provided and patient efforts made to break down evil old traditions of the unimportance of carious teeth and ear discharge, show that the enthusiasm of the mothers actually outstrips the rapidity with which clinics can be supplied.

The element of domestic misfortune has further to be considered in dealing with individual cases. The writer was once asked to report on a child with very badly discharging ears, in whose case a prosecution for neglect was contemplated. Her condition was certainly horrible, but a few inquiries showed that the mother's hands were too crippled with rheumatism to hold a syringe, the father was ill with pneumonia, and the child in question was the eldest of eight, all being under twelve! Inebriates, mental defectives, and the adherents of strange sects make up the bulk of the intractable conscientious objectors. When a child is losing her hearing because the mother is never sober enough to keep a hospital appointment, or another has high myopia whose father maintains that spectacles are contrary to Holy Writ, there are few medical officers who would not feel justified in advising their committees to prosecute.

The school doctor may also be brought into the witness-box to support a summons against a parent who is refusing to send his child to some variety of special school for which it has been certified. These incidents may be extremely annoying, for the father commonly alleges that the child is "fretting itself ill" at the thought of its new school, and the unhappy doctor finds himself held up to the Court as a bureaucratic tyrant, with a poor opportunity of self-defence. The objections advanced against mentally defective schools are comprehensible, but those affecting the cripples, blind, and deaf are usually startling exhibitions of parental affection in its most perverted form. The father of a fragile lad with tubercular hip has been known to try to expose him to all the dangers of a rough boys' school with many stairs rather than have him "disgraced" by travelling in the school ambulance. Fortunately the good sense of the magistrate is usually to be relied on for a decision in the interests of the child.

Prosecutions for non-attendance at ordinary elementary schools are common enough, but the school medical officer is not asked to intervene save in the rare cases where the parent, having alleged in defence that the child is physically unfit for school, produces a medical certificate of doubtful *bona fides*. It is of course, a strong step to dispute the validity of a certificate signed by a duly qualified medical man, but certain of the documents which are sent into Education Offices would have to be seen to be credited. It cannot be seriously doubted that a few practitioners have, let us say, an inadequate sense of responsibility in the matter, but their number is fortunately decreasing. The gentleman who gave certificates of unfitness from "tonsillitis," renewed fortnightly for a consideration of sixpence, to any child with tonsils and adenoids, is no longer on the register, and the doctor who would cheerfully "sign up" a little one for cough or sore throat without even seeing it has passed away and left few successors. Such incidents have a demoralising effect on a wide circle, and directly encourage the callous exploitation of children, so the school medical officer will have little hesitation in asking magistrates to ignore such certificates.

